# Successful Pregnancy Outcome in Women with Recurrent IVF Failure and Anti-hCG Autoimmunity: A Report of Three Cases

**DOI:** 10.1155/2016/4391537

**Published:** 2016-11-24

**Authors:** Valeria Muller, Ksenia Ob'edkova, Inna Krikheli, Igor Kogan, Irina Fedorova, Elena Lesik, Evgenia Komarova, Alexandr Gzgzyan

**Affiliations:** Department of Assisted Reproduction Technologies, FSBI D. O. Ott Research Institute of Obstetrics, Gynecology and Reproductology, Saint Petersburg, Russia

## Abstract

We report three cases of effective management of infertility in women with a history of repeated unsuccessful IVF attempts, who have developed antibodies to hCG. A novel approach to conservative treatment of immunologic reproductive failure, suggested for selected patients, included membrane plasmapheresis, combined prednisolone, and intravenous immunoglobulin therapy. No adverse side effects were observed; all cases resulted in pregnancy and subsequent life births. In order to be given an adequate efficient treatment, women with recurrent implantation failure should be suspected for autoimmune factor of infertility and its possible association with anti-hCG autoimmunity.

## 1. Objective

In the last decade, significant improvements and crucial achievements have been done in the field of assisted reproduction. However, in the majority of infertility centers, only one-third of all the* in vitro* fertilization (IVF) cycles result in life birth, while the largest percentage of patients fail to achieve successful pregnancy even after repeated attempts.

Immunologic mechanisms, involved in infertility and its subsequent treatment failure, have been the subject of discussion in current literature. The link between recurrent pregnancy loss, unexplained infertility, and autoimmune disorders was first noticed by Gleicher et al. (1989) [1]. Since then, many papers have been published on the association of reproductive failure in clinically asymptomatic patients and allo- and autoantibody positivity against different reproductive tissues and cells [2–4].

Conflicting reports have described the presence of antibodies to human chorionic gonadotropin (hCG) and its relation to female fertility and pregnancy outcome. Pathological mechanisms and immunologic pathways, involved in anti-hCG autoantibody formation in women with preserved conception or recurrent pregnancy loss have been noted previously [5, 6] but remain poorly understood. As no consensus exists, various approaches to specific treatment of patients with possible autoimmune factor of infertility have been introduced [7–10].

In the article, we present three cases of women with repeatedly unsuccessful IVF attempts, who were found positive for antibodies to hCG. We report a successful pregnancy outcome and describe the treatment scheme, which was applied for the effective management of possible autoimmune infertility in selected patients.

## 2. Case Report

### 2.1. Case  1

A 39-year-old woman with secondary infertility addressed to assisted reproduction technology (ART) department of FSBI D. O. Ott Research Institute of Obstetrics, Gynecology and Reproductology (Saint Petersburg, Russian Federation) in 2015. The patient had a medical history of three naturally conceived pregnancies, which all terminated in spontaneous abortions at 8–11 weeks of gestation. Subsequently, she could not conceive for 2.5 years and underwent full infertility evaluation. Patient had regular ovulatory menstrual cycle, normal blood hormonal profile, patent fallopian tubes (confirmed by hysterosalpingogram), and partner's satisfactory semen analysis. She had no history of venous thrombosis or autoimmune disorders. Her genetic tests (karyotyping, most common inherited thrombophilia gene mutations) did not reveal any significant abnormalities. At the time of admission to our unit, the patient had already two unsuccessful IVF attempts at another center. Therefore, before performing the next treatment cycle, it was decided to evaluate autoimmune factors possibly affecting her fertility.

No significant increase in the prevalence of antithyroid, antisperm antibodies, and antibodies against seven major phospholipids in sera were noted. Further blood analysis using enzyme immunoassay (Diatex-M, Russian Federation) revealed 2.24 units of anti-hCG antibodies (summary class G and class M immunoglobulins), which exceeded normal reference values according to manufacturer's instruction (<1 units).

Conventionally, together with hematologist, it was decided to initiate a membrane separation plasmapheresis (PA) treatment before another IVF attempt. The PA procedure was performed as described previously [11] under aseptic conditions on the PCS-2 device (Haemonetics, USA). The course included three sessions with a four-day interval starting on day 5 of menstrual cycle and ending a week before ovulation induction.

Thereafter, the patient underwent gonadotropin releasing hormone (GnRH) antagonist conventional ovarian stimulation with recombinant menopausal gonadotropins (Pergoveris, Merck Serono S.A., Switzerland) at the cycle dose of 2050 IU. A trilinear appearing endometrium with a thickness of 12.0 mm on the day of recombinant hCG trigger (Ovitrelle, Merck Serono S.A., Switzerland) was registered. Sixteen oocytes were retrieved transvaginally and conventional insemination (IVF) was performed, resulting in a double good quality blastocyst embryo transfer (ET).

During IVF protocol, the patient received additional immunotherapy: oral methylprednisolone (4 mg daily) starting on the first day of controlled ovarian stimulation (COS) and three infusions of 50 g intravenous immunoglobulins (IVIG, Intratect, Biotest Pharma, Germany): first, during stimulation; second, 2-3 days after egg retrieval; third, 5-6 days after ET.

Positive beta-hCG blood test was obtained 14 days after ET and two gestational sacs were visualized one week after the test. Prednisone was continued until 7 weeks of gestation. Underlying pregnancy went without complications, but additional courses of IVIG were repeated during the first trimester. At 32 weeks of pregnancy, the woman was admitted to hospital with spontaneous rupture of membranes. At the time, absence of fetal movements and the heartbeat of one of the twins was registered. Emergency cesarean section was performed and two newborns (1500 g and 1000 g body weight) were delivered. Unfortunately, second child's death due to asphyxia was established on the first day of life. Second newborn was healthy and, at the time of preparing the article, is 3 months old with no detected abnormalities.

### 2.2. Case  2

A 34-year-old patent with a 10-year history of primary infertility due to severe male factor (asthenozoospermia) was initially presented in 2014. During infertility investigations she was shown to be ovulating and had a normal uterus and tubal patency. As intracytoplasmic sperm injection (ICSI) was strictly indicated in her case, the woman had already completed four unsuccessful ART cycles without preceding additional infertility evaluation.

For her first attempt at our clinic, patient underwent IVF-ICSI program, showing good follicular responses to gonadotropic stimulation, but no pregnancy was achieved. For the second attempt, owing to recurrent implantation failure, we completed the treatment with IVIG. The woman conceived, however, that pregnancy resulted in spontaneous abortion in the first trimester. Therefore, before the next IVF-ICSI attempt, when the patient was already at the age of 36, a full immunological investigation was performed and the presence of anti-hCG (1.76 units) autoimmunity was established.

Concerning this founding, three PA procedures were carried out and immunosuppressive therapy with 4 mg methylprednisolone daily was started two weeks before ovulation induction. Long GnRH agonist protocol with recombinant and menopausal gonadotropin stimulation (cycle dose, 2400 IU) and recombinant hCG trigger was performed. Ten oocytes were obtained via ultrasound-guided pick-up and ICSI was carried out. After 5 days in culture, one good quality blastocyst was transferred to the uterus resulting in vial one-fetus pregnancy confirmed sonographically at 8 weeks.

As well as in previous case, IVIG was administered both during IVF protocol and after the positive pregnancy test. The patient carried the pregnancy to term without complication and gave birth by cesarean section to a healthy term neonate weighing 3200 g.

### 2.3. Case  3

A 31-year-old woman with a 4-year history of infertility was referred to IVF unit in 2014. She was considered to have combined male and tubal infertility as bilateral tubal blockage was defined previously by diagnostic laparoscopy and teratozoospermia, by sperm analysis. The patient had a history of four IVF-ICSI attempts: two ended with negative beta-hCG test results, and other two resulted in spontaneous abortions at 20 and 6 weeks of pregnancy. The woman had no history of venous thrombosis, showed no clinical symptoms of autoimmune diseases, and was negative for antiphospholipid, antithyroid, antisperm, and antiovarian antibodies.

Anti-hCG antibody positivity (2.1 units) had been first detected in patient before she underwent her first IVF-ICSI cycle with GnRH antagonist stimulation protocol at our center. Despite the adequate ovarian response to exogenous gonadotropins, good quality embryo transfer, and IVIG infusions (performed during IVF-ICSI cycle as described above), no pregnancy occurred. Therefore, after this failure, three PA procedures were carried out and oral 4 mg methylprednisolone pretreatment was started before the subsequent IVF attempt.

Long downregulation protocol with leuprolide acetate (Decapeptyl, Ferring, Germany), recombinant human follicle-stimulating hormone (Gonal-F, Merck Serono S.p.A., Italy) at the total dose of 2350 IU, and recombinant hCG trigger was performed. On day 5, after eighteen retrieved oocytes were fertilized with ICSI, two good quality blastocyst were transferred transvaginally to the uterus under ultrasound guidance. In the course of IVF treatment, we followed our scheme of IVIG supplementation.

Fourteen days after embryo transfer, serum beta-hCG was 678 IU/L; two weeks later transvaginal ultrasonography scan showed implantation of two gestational sacs with a positive fetal heart tone. Delivery by classical cesarean section was performed at the term of 36 weeks. Two healthy children (2100 g and 2300 g) were born; no adverse neonatal outcomes were noted.

## 3. Discussion

At the present time, despite the complete infertility work-up and a high quality multiple embryo transfer, there is still a substantial number of couples that will not succeed in having a child even after repeated treatment attempts. Failure to achieve a successful pregnancy following 2–6 completed IVF/ET cycles, in which ten or more morphologically good embryos were transferred into the uterus, is generally defined as a recurrent* in vitro* fertilization failure (RIF) [4, 8]. RIF reflects the lack of implantation, has a serious impact on mental and social well-being of couples, and is expectedly associated with physical, emotional, and financial distress [7]. Among potential causes of RIF, uterine factors (aberrant endometrial receptivity, immunological incompatibility) and embryo quality have been widely investigated [12]. Nevertheless, up to 20% of couples, whose IVF failures could not be explained by anatomic, endocrine, or chromosomal abnormalities, are determined to have “unexplained” infertility [3, 6].

It has been suggested that despite being clinically different disorders, both repeated reproduction failure (RIF plus early recurrent miscarriage) and “unexplained infertility” share similar characteristics with regard to immunological biomarkers in blood or endometrium [8, 12]. Contradicting data have been published regarding the influence of certain antibodies and/or general activation of immune system on preimplantation embryo, implantation process, or placenta formation [3, 7, 13]. It has been proposed that reduced fecundity might be associated with the dysregulation of immune system reactions resulting in enhancement of autoantibody production. Increased prevalence of various types of allo- and autoantibodies (antiphospholipid, antithyroid, antisperm, antiovarian, anti-zona pellucida, anti-trophoblast, antinuclear, anti-smooth muscle, anti-endometrial antibodies) were detected in patients experiencing RIF [2–4]. Other immunological parameters such as elevated numbers of peripheral blood natural killer (NK) cells, immunological defects at the T-cell level, altered levels of cytokines, and the presence of specific maternal HLA-G polymorphisms might as well be associated with implantation failure [3, 7, 8]. In our cases, due to financial reasons, patients were able to undergo only antiphospholipid, antithyroid, antisperm, antiovarian, and anti-hCG antibody evaluation. Unfortunately, general parameters of immune system activation or susceptibility to autoimmune reactions were not assessed at the point. However, blood analysis showed elevated levels of anti-hCG antibodies that helped us to suspect immunological disturbance as a cause of previous reproductive failures in the selected subjects.

Data on anti-hCG antibody prevalence in infertile women and its biological effects is limited. It is known that beta subunit of the hCG is not immunogenic in women [6]. Therefore, beta subunit of hCG has been a target for the eventual birth control vaccine since 1970s. Nowadays, the vaccine against hCG is the first and the only birth control vaccine to go successfully through Phase II efficacy trials [5]. The study of Talwar et al. provided evidence that circulating persistent anti-hCG antibodies prevented pregnancy in 1224 sexually active women. However, the authors revealed that fertility was restored when anti-hCG levels in serum dropped below 35 ng/mL and emphasized the fact that booster injections were required to maintain antibody titers. The mentioned findings enable to hypothesize that anti-hCG autoimmunity might be immunologically “boostered” by exogenous hCG administration during various infertility treatments and/or by spontaneous pregnancies [6]. Indeed, anti-hCG antibodies were reported to be detected in young male who had been treated with exogenous hCG [14]. In the subset of our patients, women either suffered from secondary infertility or had already undergone controlled ovarian stimulations during previous IVF procedures, when they were first found positive for anti-hCG immunoglobulins.

The possible role of the particular antibody in the establishment and maintenance infertility is not clearly understood. Hearn et al. reported that marmoset embryos exposed to anti-hCG immunoglobins fail to implant [15]. Immunization against hCG has been shown to block fertility in baboons and the rhesus monkeys [16]. Human anti-hCG vaccination studies provided evidence that elevated antibody levels might be the prime cause of reproductive failures. However, it is clear that not every woman will develop autoimmunity to hCG after pregnancy or infertility treatment. In this regards, structural alteration, possibly caused by a mutation in one of the beta-hCG genes and/or functional abnormalities of general immune system, should not be ruled out [6].

Nowadays, it still remains a major challenge for both clinicians and researchers to improve ART outcomes in women with RIF. As dysregulated pathways remain largely unknown, diverse immunomodulatory interventions have been suggested as supplementary to IVF treatment for this cohort of patients [7, 8]. In our cases, as all women were suspected to have autoimmune factor of infertility, probably also associated with anti-hCG autoimmunity, the decision to apply immunomodulation therapy was taken. The treatment course, which resulted in successful life birth, included membrane plasmapheresis, intravenous immunoglobulins and glucocorticoids [11].

Plasmapheresis is commonly used for treatment and prevention of acute viral infections. In gynecology and reproduction, PA is applied to women with tubal infertility and peritoneal endometriosis and in cases of ovarian hyperstimulation syndrome [11]. The detoxifying effect of PA is achieved not only by mechanical removal of biological toxins, inflammatory mediators, and metabolic products, but also by stimulation of natural immune mechanisms. In our cases, we decided to perform PA course before IVF treatment for the purpose of both mechanical elimination of anti-hCG antibodies and the modulation of immune system functioning.

Another immunotherapy, described in the present report, is glucocorticoid treatment. Corticosteroids (prednisolone and methylprednisolone: FDA category C) are the most effective and secure pharmacological group to decrease the activity of autoimmune disease even in pregnant patients [8]. In current literature, the use of glucocorticoids in IVF/ICSI cycles for women with RIF or “unexplained” infertility has been described in numerous heterogeneous trials. A Cochrane database meta-analysis by Boomsma et al. [9] showed borderline statistical significance (no clear evidence) that peri-implantation administration of glucocorticoids in IVF, but not in ICSI cycles, was associated with an improvement in clinical outcome. In our unit, for women with RIF we give low doses of methylprednisolone (4 mg) shortly before IVF protocol and continue the therapy either up to the day of positive pregnancy test or until clinical pregnancy establishment.

Another treatment drug, used in the described cases, was intravenous immunoglobulin. IVIG, being a monomeric immunoglobulin class G preparation found in human blood, has been applied for more than 50 years as a therapy of immunodeficiency disorders, such as Kawasaki disease, multiple sclerosis, and dermatomyositis [7, 10]. Intravenous immunoglobulin, due to its anti-infective, anti-inflammatory, and immunoregulatory properties, has been introduced empirically to ART programs with the aim of improving IVF success. The effectiveness of IVIG in women with RIF has been evaluated in several prospective, randomized, placebo-controlled trials and summarized in two meta-analysis [8, 10]. Both of them showed that in patients experiencing “unexplained” repeated IVF failure and/or “unexplained” infertility the use of IVIG was significantly associated with a higher clinical pregnancy and lower miscarriage rate. However, in the most recent of the two of meta-analysis by Nyborg et al. of a total of 8207 participants [8], authors did not reach statistical significance for the live birth rate per ET. It should be noted that significant variations could be found between the authors regarding the applied schemes of IVIG therapy: some researchers do adjustment according to body weight (400–500 mg/kg); others apply a fixed IVIG dosage of 20–25 g. Timing of IVIG infusion differs as well between the papers: injections start at the time of COS, ET, or on the day of the known pregnancy and are maintained until 7 or 28 weeks of gestation [8, 10]. At our ART department, we use triple 50 g IVIG infusions during IVF protocol: first, during ovarian stimulation; second, 2-3 days after oocyte pick-up; third, 4-5 days after embryo transfer.

In summary, the presence of anti-hCG autoimmunity should be suspected even in patients with a known factor of infertility, who have RIF following IVF treatment. Autoimmune system dysfunction should be considered in these cases when planning an ART cycle. We support the hypothesis that immunotherapy might be beneficial for women with repeated IVF attempts. To our knowledge, this is the first study to discuss complex immunomodulating therapy including membrane plasmapheresis, prednisolone, and intravenous immunoglobulins for successful and safe treatment of patients with RIF. However, larger prospective controlled studies are required to confirm these findings and determine diagnostic options, as well as a unified treatment scheme, for women with recurrent reproduction failures.
